# Glymphatic System and Mitochondrial Dysfunction as Two Crucial Players in Pathophysiology of Neurodegenerative Disorders

**DOI:** 10.3390/ijms241210366

**Published:** 2023-06-20

**Authors:** Kamila Kopeć, Stanisław Szleszkowski, Dariusz Koziorowski, Stanislaw Szlufik

**Affiliations:** Department of Neurology, Faculty of Health Sciences, Medical University of Warsaw, 02-091 Warsaw, Poland; kopec.kaamila@gmail.com (K.K.); szleszkowski@gmail.com (S.S.); dariusz.koziorowski@wum.edu.pl (D.K.)

**Keywords:** neurodegeneration, glymphatic system, mitochondrial dysfunction, Alzheimer’s disease, Parkinson’s disease, sleep disorders, neuroinflammation

## Abstract

Neurodegenerative diseases are a complex problem affecting millions of people around the world. The pathogenesis is not fully understood, but it is known that both insufficiency of the glymphatic system and mitochondrial disorders affect the development of pathology. It appears that these are not just two independent factors that coexist in the processes of neurodegeneration, but that they often interact and drive each other. Bioenergetics disturbances are potentially associated with the accumulation of protein aggregates and impaired glymphatic clearance. Furthermore, sleep disorders characteristic of neurodegeneration may impair the work of both the glymphatic system and the activity of mitochondria. Melatonin may be one of the elements linking sleep disorders with the function of these systems. Moreover, noteworthy in this context is the process of neuroinflammation inextricably linked to mitochondria and its impact not only on neurons, but also on glia cells involved in glymphatic clearance. This review only presents possible direct and indirect connections between the glymphatic system and mitochondria in the process of neurodegeneration. Clarifying the connection between these two areas in relation to neurodegeneration could lead to the development of new multidirectional therapies, which, due to the complexity of pathogenesis, seems to be worth considering.

## 1. Introduction

Neurodegenerative disorders are a heterogeneous group of progressive disorders, from which the two most common diseases are Alzheimer’s disease (AD) and Parkinson’s disease (PD). They are a major health problem worldwide, particularly in older adults. Progression of the disease leads to a deterioration in the quality of life, disability, and ultimately death of millions of people affected by these diseases. Alzheimer’s disease affects approximately 24 million people worldwide [1]. Due to their prevalence and lack of available causal therapy, neurodegenerative diseases pose a significant socioeconomic challenge, and finding causal therapy is a crucial and equally urgent challenge for global health care. Understanding the molecular mechanisms underlying pathogenic processes can lead to the improvement of currently available therapeutic options, the effectiveness of which is often severely limited. The discovery of new causal therapeutic approaches would definitely be a breakthrough, but so far, it has not been possible to clearly determine the etiology of disorders in this group [2]. It is known that both environmental and genetic factors can lead to their occurrence. The pathogenesis also remains not fully understood. A characteristic feature of the diseases is a pathological accumulation of proteins and the progressive loss of specific groups of neurons resulting from the presence of protein aggregates and their toxic effects [2]. Disturbances in the functioning of the relatively recently described glymphatic system are increasingly mentioned as an element of the pathogenesis of proteinopathy [3].

The brain, which is only approximately 2% of total body mass, uses about 25% of the glucose and 20% of the oxygen required by the human body [4]. The brain is, therefore, one of the organs with the highest level of metabolism, which entails a high production of metabolic wastes. Maintaining homeostasis within it is a key and, at the same time, a very demanding task in which the forementioned glymphatic system plays a role [5]. Many substances like beta-amyloid (Aβ) and tau protein removed via the recently discovered glymphatic system have a potentially toxic effect on the cells of the central nervous system (CNS) [6,7]. Glymphatic clearance is based on the bulk flow of cerebrospinal fluid (CSF) [8]. CSF is mostly produced in the choroid plexuses of the ventricular system of the brain in the amount of 430–530 mL per day [9].

Its one-way flow in the glymphatic system can be distinguished into three main stages [6]. Firstly, CSF from the subarachnoid space enters the brain parenchyma along periarterial spaces. Subsequently, interstitial space mixes with interstitial fluid and solutes. A crucial role in flow to the interstitial space is played by astrocytes and Aquaporin-4 (AQP4) water channel expressed in high density on their endfeet surrounding the vessels [8]. AQP4, which is a molecule commonly found in the brain, is a particularly important component of the glymphatic system [8,10]. Interestingly, AQP4 abnormalities have been shown to be observed in neurodegeneration [11]. Perivascular localization of AQP4 is disturbed in AD patients [12]. Moreover, it has been shown that the progression of cognitive impairment in AD may be related to variations in the AQP4 gene [13]. Finally, interstitial fluid drains towards the perivenous spaces to be removed from the brain [6,8]. It is significant that the glymphatic system is most active during sleep [14]. One of the factors affecting this state of affairs is that the sleep–wake state determines the volume of the interstitial space. In an animal model study, it has been shown that with the state of sleep, whether natural or anesthesia-induced, the interstitial space increases by 60%. This, in turn, has a direct impact on the significant intensification of the exchange of CSF with interstitial fluid [15]. Flow through the described spaces is induced by many factors. The aforementioned state of natural or anesthetic sleep and expression level and polarization of AQP4 appear to be essential for the glymphatic flow. However, there are other factors affecting glymphatic clearance, which may include: arterial pulsatility, respiration, CSF production, and body position [16]. In a study on an animal model, it was shown that a 27% reduction in the vessel wall pulsatility of intracortical arterioles is associated with an impaired glymphatic flow and, thus, Aβ drainage. Importantly the reduction in arterial pulsation is commonly seen in aging brains [17]. Similarly, a feature of the aging brain is reactive astrogliosis, which is a process associated with AQP4 abnormalities that entails glymphatic flow impairment [17,18]. This is consistent with the fact that neurodegenerative diseases are most common in the elderly [1]. The aforementioned sleep, or rather its disorders, is an inseparable element of many neurodegenerative diseases [19,20,21,22]. All these events seemed to indicate a cascade of damage affecting the glymphatic system. Then, there is also an increase in the incidence of neurodegenerative diseases [1]. Continuous rotation and, thus, the possibility of constant CSF flow, seems to be crucial for glymphatic system efficiency. Any situation that disrupts production or the flow itself may, at some point, potentially lead to the impairment of its drain waste function [8,23]. Similarly to mitochondriopathies, it seems that impairment of glymphatic function may play an important role in the pathogenesis of neurodegenerative disorders [24].

Mitochondria are cellular organelles necessary for the functioning of eukaryotic cells. Mitochondria are responsible for orchestrating cellular energy production and cellular processes, including cell cycle control and cell death [25]. Considering how high the energy demand of the brain is and how intense the ATP production process must be, it is reasonable that the disturbance of this process will result in pathological changes in the brain and that the neurons are particularly vulnerable to mitochondriopathies [26]. Mitochondria contain mtDNA encoding proteins necessary in the process of oxidative phosphorylation (OXPHOS) [27]. In the OXPHOS process, they generate the ATP crucial for cell functioning, and reactive oxygen species (ROS) are produced as a by-product in this process. ROS, which are highly reactive molecules, could present a toxic effect on CNS cells [28]. In a healthy brain, ROS formation is in balance with the rate of neutralization [27]. The role of mitochondria in neurodegenerative processes has been known for years. When the balance between the production and neutralization of ROS is disturbed, oxidative stress occurs, which is directly related to cell damage and neurodegeneration [29]. Excessive accumulation of ROS in patients with AD may increase mitochondrial dysfunction, and oxidative stress intensifies the accumulation of Aβ. What is more, Aβ itself increases oxidative stress and leads to mitochondrial dysfunction, which closes processes in a vicious circle [30]. In PD, oxidative stress will lead to damage in a vicious circle similar to AD. Primary mitochondrial abnormalities generate oxidative stress that leads to further damage and exacerbates pathological processes [31]. Oxidative stress plays a particularly important role in the degeneration of dopaminergic neurons, which are frequently exposed to it in PD [32]. ROS are also signaling molecules that mediate in the signaling pathways. Prolonged oxidative stress leads to the activation of signaling pathways, which results in the promotion of an inflammatory response [33]. Activation of these pathways leads to the promotion of an inflammatory response via the activation of microglia, activation of inflammasomes, and to the secretion of proinflammatory molecules such as cytokines these events [33]. Damage-associated molecular patterns (DAMPs) are molecules that trigger the cascade leading to the expression of inflammation mediators. As a result of damage to the mitochondrial membranes, DAMPs may be released, which, apart from ROS, is also mtDNA. DAMPs are recognized by microglial receptors, which activation leads to an inflammatory response [34]. It should be emphasized that all these processes do not occur independently, but they interact in the process of neurodegeneration and drive each other [35]. The purpose of this review, however, is not to analyze the mitochondrial disorders themselves in relation to neurodegeneration, but to emphasize the possible correlation with the glymphatic system in neurodegenerative processes. This seems particularly interesting because both of these areas are involved in pathogenesis [24,36]. Looking at their functioning as closely related systems may indicate new directions of research and possibly new therapeutic models.

## 2. Correlation of Mitochondrial and Glymphatic Dysfunction in the Accumulation of Aggregates

Degenerative diseases are often called proteinopathies. This is due to the fact that in their course, there is an accumulation of protein deposits in the brain, which both directly and indirectly may play a significant role in the disease process. In PD, the protein whose accumulation entails many consequences is alpha-synuclein (α-Syn), while in AD, the main pathologically accumulating protein is Aβ [2,37]. It is well known that dysregulated mitochondrial homeostasis contributes to the process of formation and accumulation of toxic aggregates [3]. Vice versa, the glymphatic system in a healthy brain is involved in the clearance of waste products from the CNS [38]. The question arises as to what processes the glymphatic system and mitochondrial metabolism can jointly contribute to the progression of the disease, and whether possible therapeutic procedures should simultaneously focus on improving the efficiency of the glymphatic system and compensating for neuroenergetic impairment. α-Syn is a protein that is normally expressed in the brain and is involved in synaptic processes in the neurons [39]. Mitochondria isolated from PD patients have a relatively much higher content of α-Syn [36]. Accumulation of α-Syn is associated with its aggregation and the acquisition of a toxic function [40]. Mitochondrial dysfunction and synucleinopathy interact in the pathogenesis of PD. α-Syn leads to impairment of ATP production and inhibits complex I. What is more, it leads to depolarization of the mitochondrial membrane, which results in increased production of ROS [41]. Furthermore, the oxidative environment promotes oligomerization. A positive-feedback loop develops in which ROS promotes oligomerization of α-Syn, which impairs complex I function and drives increased ROS generation. All of these events lead to further oligomerization [41]. Moreover, α-Syn appears to have a preferential affinity for mitochondria over other organelles [36]. The bidirectional interaction of mitochondria with protein aggregates is also characteristic of AD [37]. The linkage of Aβ aggregates and mitochondria is explained in the mitochondrial cascade hypothesis [42]. Its detailed wording is still under discussion, but at the moment, it is assumed that oxidative stress, along with mitochondrial dysfunction, plays a significant role in the pathogenesis of the disease. Aβ causes mitochondrial structural changes and disruption of the electron transport chain and bioenergetics defects. Furthermore, dysregulated mitochondrial homeostasis results in increased production of ROS, leading to oxidative stress [42]. Again, we can define this as a positive feedback loop in which Aβ promotes oxidative stress, and oxidative stress is involved in the promotion of Aβ deposition [43]. Long before the discovery of the glymphatic system, it was obvious that sleep plays a significant role in the pathogenesis of neurodegenerative diseases [44]. The specific and complex role of sleep in relation to mitochondria is discussed in the next paragraph, but here the focus is on glymphatic clearance itself. It has been proven that the activity of the glymphatic system increases during sleep, and the increased glymphatic flow at this time allows the removal of waste products, including protein deposits, which seems to be very important. Thus, in the pathogenesis of neurodegenerative diseases, the inefficiency of the glymphatic system seems to be crucial [14]. When we add to this the previously described bidirectional dysfunction of mitochondria and protein aggregation, impaired glymphatic clearance appears to be another factor exacerbating the situation. Many publications emphasize the impairment of glymphatic clearance in neurodegeneration [3,45]. The study on animal models revealed that when a fluorescent β1–40 amyloid was injected into the striatum of mice, it was removed from the brain via the glymphatic paravenous efflux pathway. Moreover, the knockout of AQP4 reduced amyloid β1–40 clearance by 55% [7]. Importantly, it seems that Aβ itself can interfere with the flow in the glymphatic pathway in a feedback mechanism [24]. Using a PET scan, it was shown that in patients with AD, ventricular CSF clearance was inversely associated with amyloid deposition [46]. In patients with AD in relation to healthy ones, the postmortem studies showed abnormalities in the expression and localization of AQP4 [24]. The process of AQP4 mislocalization, Aβ accumulation, and the impact of these processes on glymphatic clearance seems to be closely correlated, but this area requires further research. There are analogous premises regarding the coexistence of glymphatic disorders and α-Syn aggregation in PD. An animal model study showed that decreased AQP4 expression leads to further accumulation of α-Syn and impairs glymphatic flow [47]. However, overexpression of α-Syn may lead to reactive astrogliosis, which impairs AQP4 polarity and its removal by glymphatic clearance [48]. The participation of mitochondrial and glymphatic disorders in the deposition of toxic aggregates in neurodegeneration seems probable. However, the problem is very complex and multidirectional.

**Figure 1:**
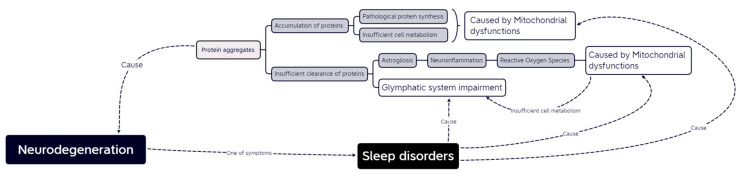
Possible correlation of mitochondrial dysfunction and glymphatic system impairment in neurodegeneration.

At the moment, it seems impossible to determine the beginning of the cascade of events that seem to lead to the progression of the disease. The role of mitochondriopathies and glymphatic system disturbance seems to be on two ends of neurodegenerative disorder progression, but the relation between these two looks much closer when factors more related to PD and AD are taken into consideration. The interaction between mitochondrial and glymphatic activity may represent an important point of convergence in disease pathogenesis (Figure 1). Understanding the mechanism of the relationship could stimulate the development of new therapeutic approaches.

## 3. Mechanisms Linking Glymphatic System and Mitochondriopathies with Sleep and Neurodegeneration

The connection between sleep and neurodegenerative disorders has been emphasized in many studies [19,20,21,49,50]. Patients with Alzheimer’s disease suffer from such typical symptoms of sleep disorder as difficulty in falling asleep, arousal at night, repeated awakenings, and waking up too early in the morning [19]. Moreover, it has also been proven that during AD progression, sleeping disorders worsen [20], and their intensification is used as a predictive factor of mortality in the last stages of the disease [21]. Patients with Parkinson’s disease also suffer from sleeping disturbances caused by motor and non-motor symptoms [22]. These include restless leg syndrome (RLS), obstructive sleep apnea (OSA), rapid eye movement sleep behavior disorder (RBD), and circadian rhythm disturbance [51,52,53]. All of them can lead to insomnia and reduced sleep quantity and quality. Sleep disorders have not only been shown to coexist with neurodegenerative disorders, but several scientific articles demonstrate their effect on specific components of the CNS. One of these components affected by sleep disturbance, which has been described repeatedly, is the glymphatic system [19,20,21,22]. It is known that the glymphatic pathway function is a feature of the sleeping brain. During sleep, the intensity of glymphatic system activity increases rapidly, just as during anesthesia [8]. In a mouse study, it was shown that the CSF influx in the awake state was reduced by 95% compared to sleeping mice [15]. Human studies also indicate that sleep is associated with greater glymphatic clearance compared with wakefulness [54]. Sleep disturbances are relatively common in elderly people [55]. This may, to some extent, explain the fact of declining glymphatic transport efficiency in aging, which is highlighted in the literature [16] which, in turn, may be related to the increased incidence of neurodegenerative diseases in this age group. Another example of a sleep disorder showing a significant link between neurodegeneration and the glymphatic system is RBD, which was previously mentioned as a common symptom of PD. Patients with RBD are known to show disruption of normal sleep architecture, and a recent meta-analysis found that the estimated risk for RBD patients to develop a neurodegenerative disease over a long-term follow-up is more than 90% [56]. Moreover, research indicates the presence of glymphatic-system dysfunction in patients with RBD [57]. Since sleep disorders impair the functioning of the glymphatic system, and thus reduce cerebral fluid clearance, the role in the pathogenesis of neurodegenerative disorders seems very probable. Reduced glymphatic flow results in lowering waste product removal and increases the risk of the formation of protein aggregates characteristic of a disease entity and may contribute to the development of disease [3]. Regarding these facts, accumulating evidence indicates that sleep disorders which are characteristic symptoms of many neurodegenerative diseases such as PD and AD, may potentially contribute directly to the pathogenesis rather than simply being symptoms (Table 1) [58]. However, many years before describing the glymphatic system and its dependence on sleep, it was known that mitochondrial disorders are a key factor in the pathogenesis of neurodegenerative diseases [59].

Articles can be found in the literature describing the effects of sleep disorders on mitochondrial function [60,61,62]. It has been established in mouse studies that chronic sleep restrictions cause morphological changes in the mitochondria of the frontal cortex, lower ATP levels, reduced cytochrome c oxidase concentration, and decreased mitochondrial membrane potential [60]. Moreover, mitochondria-related Aβ accumulation was significantly higher in mice after chronic sleep deprivation (SD) [60]. Another study compared chronic sleep deprivation between Alzheimer’s disease-like pathology—*AβPPswe/PS1 ΔE9* transgenic mice (TG), wild-type mice (WT), and non-sleep-deprived (NSD) control mice. It revealed that chronic sleep deprivation caused significant mitochondrial damage, caspase cascade activation, and neuronal apoptosis in the hippocampus of both TG and WT mice. After 2 months of SD in both TG and WT, altered Aβ protein precursor processing and an elevated level of phosphorylated tau protein were found. In addition, TG mice after SD showed more amyloid-β1-42 production and developed more senile plaques in the cortex and hippocampus than NSD-treated TG mice. It provides empirical evidence that chronic SD is not only a risk factor for Alzheimer’s disease, but also enhances its progression [63]. One rat study focused solely on 72 h rapid eye movement sleep deprivation effect on mitochondrial biogenesis in the hippocampus. It showed that RBD significantly increased the mtDNA copy number in the hippocampus and also increased the expression of cytochrome c oxidase subunit 4I1. The authors concluded that REM–SD may induce mitochondrial dysfunction in the brain [61]. These findings make it possible to link mitochondrial dysfunction, glymphatic dysfunction, and the progression of neurodegenerative diseases, as all three are caused by REM–SD. Human studies have also made some connections between mitochondrial disorders and sleep disturbances. One article suggests that 70% of individuals with mitochondrial optic neuropathies have complaints about their sleep quality [64]. Another human study revealed that individuals with primary insomnia have increased malondialdehyde enzyme activity—an oxidative stress marker, and also decreased activity of two main markers of antioxidative properties—glutathione (GSH) and glutathione peroxidase [65]. A recent meta-analysis examined the results of animal and human studies on mitochondria’s role in sleep. It established that both reached a consensus on two points. First, chronic sleep deprivation, including REM–SD, causes abnormalities in mitochondria morphology and bioenergetics. Second, sleeping disorders cause increased concentration and accumulation of ROS markers and reduction of numerous antioxidants [62].

The proper mitochondrial function also appears to be crucial for the efficient functioning of the glymphatic system. This has been demonstrated by studies that have focused on its basic function. The mechanism of the glymphatic system is based on the flow of CSF. It is known that approximately 75% of CSF is produced in the choroid plexuses of the ventricular system of the brain [66]. This is achieved by generating an osmotic gradient that entails water flowing across the epithelium to the lumen of the ventricle, thereby producing CSF [8]. However, the formation of an osmotic gradient is the final stage of CSF production. Before that, many processes allow for its creation, and the activity of Na^+^/K^+^-ATPase seems to be crucial for all of them. Na^+^/K^+^-ATPase is localized in the apical membrane of the choroid plexus epithelial cells. Their pivotal role in the production of CSF is proven by studies that inhibit Na^+^/K^+^-ATPase, which resulted in CSF production being reduced by 50–60% [8]. Importantly, the decrease in CSF production occurs in the course of AD [67] and is also characteristic of the aging process when CSF production is decreased [68]. However, Na^+^/K^+^-ATPase activity requires significant amounts of ATP to function [69]. So, the energy required for CSF production comes largely from Na^+^/K^+^-ATPase activity. In comparison, that supply of energy comes from OXPHOS in the mitochondria and glycolysis. Disturbance of energy homeostasis in the CNS may affect CSF production. As is commonly known, mitochondria play a central role in energy metabolism, regulation of apoptosis, and maintenance of redox balance [25]. Mitochondrial dysfunction is considered one of the causal factors in the pathogenesis of neurodegenerative disorders [70]. Many studies raise the issue of bioenergetic impairment in neurodegeneration. One of them showed that exposure to α-Syn decreased mitochondrial basal oxygen consumption rate and respiratory capacity in vitro [71]. There is also compelling evidence for mitochondrial complex I inhibition in PD [72]. Moreover, reports suggest cytochrome c oxidase activity in AD subjects is 10–50% less than in age-matched controls [73]. Thus, the bioenergetics of the CNS is undoubtedly disturbed in neurodegeneration. The question arises of how this frequent neurodegenerative disease defect in mitochondrial energy metabolism can affect the CSF production and glymphatic system. It remains to be clarified how abnormalities in the functioning of both these processes maintaining brain homeostasis in healthy people affect each other in neurodegeneration, and how they together affect the course of the disease.

In the course of neurodegenerative diseases, there is no doubt that metabolism deviates from homeostasis [74]. The aforementioned Na^+^/K^+^-ATPase is responsible not only for the production of CSF, but also for the maintenance of neuronal excitability, and conduction of the action potential, which is crucial for the functioning of the CNS [75]. Thus, the impact of energy disruption and the resulting ATP deficiency would not only impair CSF production, but would potentially be disastrous in its effect on the entire CNS. Maintaining the production of ATP at a sufficient level in conditions of impaired mitochondria, therefore, requires some kind of compensation. The survival of the nerve cell, especially under conditions of impaired metabolism, depends on maintaining sufficient ATP production and ROS protection, whose production increases in such a condition. In neurodegenerative diseases such as AD and PD, where the bioenergetics is disturbed, there is a decrease in the level of ATP derived from OXPHOS and an increase in ROS [76]. Therefore, there is a compensatory intensification of a process called aerobic glycolysis. Aerobic glycolysis is a phenomenon often mentioned as associated with the state of neuronal degeneration and mitochondrial impairment [76]. There is a transition from ATP production in the TCA and OXPHOS cycle to glycolysis despite the availability of aerobic conditions [77]. Probably, the upregulation of glycolysis in neurons may act as a compensatory mechanism in response to mitochondrial dysfunction [78]; thus, glycolysis rises above the physiological state in both AD [79] and PD [80]. In aerobic glycolysis, pyruvate is converted to lactic acid by lactic acid dehydrogenase-A [81]. For decades, the role of lactate in the functioning of the brain has been considered. The astrocyte-neuron lactate shuttle (ANLS) model indicates its role as a substrate for OXPHOS occurring in neurons [82]. In addition, lactate provides signals that modulate neuronal functions, including excitability, plasticity, and memory consolidation [83]. Research suggests that energy metabolism and its dependent lactate levels may also interact with the glymphatic system in regulating the sleep-wake cycle. Fluctuations in lactate levels coincide with typical activity of the glymphatic system. The concentration of lactate in the brain increases during wakefulness when glymphatic clearance is reduced [84]. However, during sleep, when glymphatic activity increases, lactate concentration decreases. The exceptionally high correlation between increased lactate during wakefulness and decreased lactate during sleep assumes that elevated lactate may underlie some component of sleep homeostasis [84]. Dynamic changes in brain lactate level are regulated by the lymphatic clearance functions played by the glymphatic system. Data suggest that the glymphatic system, which is activated by natural sleep or anesthesia, flushes excess lactate out of the brain [85]. Furthermore, studies in which the impairment of the functioning of the glymphatic system, e.g., through the deletion of AQP4, decreases the decay in brain lactate, show similar outcomes [86]. Dysregulation of neuroenergetics, lactate production, and ATP in neurodegenerative diseases, and the fact that the glymphatic system is responsible for sleep-wake cycle changes in brain lactate concentration indicate a possible interdependence of these components.

The connection between mitochondriopathies, the glymphatic system, and neurodegenerative diseases in the context of sleep disorders seems very promising. However, it is worth noting that one more factor links them all together—melatonin.

Melatonin is a molecule that can be seen in high concentrations in many cells throughout the body. In the human body, there are two different pools of melatonin; one, called pineal, is synthesized by the pineal gland and can be found in blood and CSF, and the second, called extrapineal, is synthesized in different tissues and is present in them [87]. For many years, melatonin has been known for its sleep-promoting and sleep-maintaining properties and for resetting the circadian cycle [88,89,90,91,92]. Although melatonin is a molecule that has accompanied the evolution of life on earth for a very long time, it was first seen to appear when the process of endosymbiosis occurred [93], which is the beginning of the existence of mitochondria [94]. The relationship between the two is not only historical, but recent studies have emphasized that melatonin plays a key role in the control of proper mitochondrial functions [95,96,97,98]. Its new role is mostly recognized as having a very powerful effect as a free radical scavenger and antioxidant [99]. That melatonin is very widely present in body tissues has been proven by the discovery of the synthesizing enzymes arylalkylamine N-acetyltransferase (AANAT) and acetylserotonin O-methyltransferase (ASMT) in almost all human tissues [100]. Those enzymes were also found in the mitochondria of brain cells, which established these organelles as important for melatonin synthesis [101]. The melatonin function as a free radical scavenger appears to be most important in mitochondrial-related neuroprotective properties. It has been proven that increasing the concentration of melatonin inhibits mitochondrial DNA damage that is potentially caused by ROS [95]. Moreover, in one animal study, the administration of melatonin increases the activity of glutathione peroxidase, which is one of the main antioxidants [96]. Subsequent scientific studies further highlight the remarkable importance of melatonin in mitochondria. In some of the most metabolically active cells-hepatocytes and neurons-mitochondrial melatonin concentrations were significantly higher than in plasma and other cell types. This shows that higher levels of melatonin are found in those tissues that produce the highest amounts of ROS due to mitochondrial oxidative metabolism [97,98,102]. It was recently discovered that melatonin promotes mitophagy [103], a process of controlled removal of damaged and aged mitochondria [104]. It appears to be one of the most important melatonin functions because an imbalance between mitophagy and ROS production accelerates the aging process and leads to neurodegenerative diseases like AD and PD [105]. Melatonin affects not only the mitochondria of neurons, but also the mitochondria of astrocytes, which are a major component of the glymphatic system. It has been shown in a study on stroke model cells that melatonin administration protects endothelial cells through ApoE in a state of oxygen and glucose deprivation-reoxygenation [106]. Melatonin’s potential therapeutic effect has been established in many studies [107,108,109]. It has been shown that melatonin modulates the levels of several proteins pivotal to Aβ accumulation: ADAM10, BACE1, PIN1, and GSK3, resulting in diminished production of Aβ and elevated Aβ clearance via the glymphatic system and BBB transportation [107]. In a different study, melatonin enhanced Aβ clearance in a transgenic mouse model of amyloidosis [108]. Another recent study has shown that melatonin level is much higher in the CSF of the third ventricle than in the blood. After melatonin enters the subarachnoid and Virchow-Robin spaces, it is taken to neural tissue, where its antioxidant and anti-inflammatory properties indicate pathogenic toxins such as Aβ [109].

In summary, melatonin links the progression of neurodegenerative diseases, mitochondrial disorders, and the correct functioning of the glymphatic system. Its therapeutic effects in in vitro and in vivo studies allow us to consider new approaches to the treatment of neurodegenerative disorders.

## 4. Neuroinflammation

The role of inflammation in neurodegenerative diseases and its connection with the glymphatic system and mitochondrial function is very complex. It is known that neuroinflammation and neurodegeneration are mutually propelling processes [110]. When discussing neuroinflammation, microglia cannot be overlooked. Their physiological function, which consists of limiting possible infections and removing dead cells, is crucial for maintaining brain homeostasis [111]. However, the protective role in a healthy brain can become neurodestructive when the balance of action is disturbed, which is what happens in the course of neurodegenerative diseases. Persistent activation of microglia contributes to the generation of oxidative stress and neuroinflammation [112]. Although microglia are listed as the main producers of ROS, their direct synthesis takes place in the mitochondria. ROS are formed as a by-product of OXPHOS [112]. Unfortunately, one of the main areas of the toxic impact of ROS is their producers-mitochondria. This leads to a self-perpetuating process of neuroinflammation as mitochondrial damage induces released mitochondrial constituents. These molecules, in particular mtDNA, as damage-associated molecular patterns (DAMPs), trigger a danger signaling response and intensify the inflammatory response [113]. The importance of inflammation and the coexistence of mitochondrial dysfunction in PD and AD is widely described in the literature [114,115]. Mitochondria, as the main producer of ROS, plays an important role in the activation of the NLRP3 inflammasome, which can lead to inflammasome activation. This happens by, among other things, impairing the work of the complexes of the mitochondrial respiratory chain [116]. NLRP3 activation, however, leads to the caspase-1-dependent secretion of proinflammatory cytokines such as interleukin-1β and IL-18, followed by lytic cell death [117].

Some neurodegenerative diseases are significantly associated with the activation of inflammasome [118]. In PD, it has been reported that dopamine deficiency restricts NLRP3 inflammasome activation [119]. NLRP3 inflammasome deregulation is also found in cells with a mutation in the *Parkin* gene [118], which is the second most commonly known cause of PD [120]. Moreover, inflammasome-associated α-Syn aggregation has been described [121]. Similar connections can be found in AD studies that report that Aβ activates NLRP3 inflammasomes and macromolecules that are involved in this process, further intensifying its aggregation [122]. Thus, there are many arguments supporting the importance of the correlation between mitochondrial impairment and the development of neuroinflammation in neurodegenerative disorders. What is particularly important is that it seems likely that increased NLRP3 inflammasome activity co-occurs with microglial activation and impaired glymphatic clearance of Aβ [123].

It is known that microglial activation contributes to neuronal damage in neurodegenerative diseases [124]. This condition is observed in both AD [125] and PD [126]. Increased ROS production and proinflammatory mediators have been associated with the inflammatory activation of microglia. Unfortunately, these products can lead to damage to the mitochondria, which seem to be particularly sensitive to their effects. One of the effects of their interaction may be a mutation of mitochondrial DNA and enzymes of the mitochondrial respiratory chain [127]. Moreover, dysfunction of mitochondria can induce microglial activation [127]. Importantly, it has been shown that active microglia can induce reactive astrocytes, which gain a neurotoxic function and can lead to cell death [128].

Astrogliosis is a term used to describe a change in astrocyte activity in response to pathological events in the CNS, such as injury or disease [129]. Astrocyte function in neuroprotection is impaired during chronic neuroinflammation. Microglia and astrocyte activation, and the release of proinflammatory mediators, lead to astrocytic hypertrophy and chronic inflammatory responses. Furthermore, these processes can drive each other in a positive feedback mechanism. All these events lead to the functional impairment of CNS cells [130]. The presence of astrogliosis not only affects the process of neurodegeneration and neuroinflammation, but also impairs the functioning of the glymphatic system itself. Astrocytes play a key role in the glymphatic system, so a disturbance in their function can lead to a significant impairment of glymphatic clearance [8]. Probably the reactive astrogliosis reduces brain clearance, thereby aggravating inflammation. It is a glymphatic impairment that may decrease the removal of proinflammatory molecules from the brain [131]. Astrocytes show polarized expression of AQP4. The highest concentration of AQP4 occurs on the endfeet of astrocytes in the immediate proximity of the vessels [8]. Impaired astrocyte function may therefore lead to impaired glymphatic clearance. Aberrant AQP4 polarity impairs CSF flow from the perivascular spaces to the parenchyma [132]. Loss of perivascular AQP4 polarization may contribute to the impairment of glymphatic pathway function. Such a situation occurs in conditions characterized by chronic neuroinflammation [131]. Loss of perivascular AQP4 polarization can be observed, among others, in aging brains [17]. This explains, to some extent, the decrease in glymphatic clearance in the elderly.

Very recent research has further advanced our understanding of the extremely important role of the glymphatic system in neuroinflammation. A groundbreaking paper was published in January 2023, demonstrating the existence of the fourth cerebral meninges, the SLYM. Its function is, among other things, to control the passage of immune cells into the inner subarachnoid space. The authors concluded that mechanical disruption of the SLYM after traumatic brain injury causes prolonged neuroinflammation and prolonged suppression of glymphatic flow [133].

## 5. Discussion

Neurodegenerative diseases like Alzheimer’s and Parkinson’s disease are one of the major challenges of modern neurology. Research has shown that mitochondriopathies or disorders of the glymphatic system may be the main causes of degenerative disease progression [3,24,45,127]. In this review paper, it has been demonstrated that these pathologies do not merely coexist with, but influence each other. The link between them is highlighted in the context of other known causes of neurodegenerative diseases.

The accumulation of protein aggregates characteristic of neurodegeneration appears to be one of the primary points at which the mitochondria and the glymphatic system converge. Aggregate formation and mitochondrial impairment have a bidirectional effect on each other, and also, directly and indirectly, affect glymphatic clearance [36,42,45]. This is due to, among other things, ROS, whose increased production also occurs in the process of neuroinflammation [41].

The role of inflammation in relation to protein aggregation and the functioning of the glymphatic system is emphasized. Mitochondria, as the main producer of ROS, can cause NLRP3 inflammasome activation [116]. Inflammasome deregulation is found in PD and AD, and, of particular importance, it is reported that increased NLRP3 inflammasome activity co-occurs with impaired glymphatic clearance of Aβ [118,123]. Similarly, the effect of the inflammatory process on astrocytes, which plays a key role in the glymphatic system, seems to be noteworthy. Disturbance of their function can lead to a significant impairment of glymphatic clearance [132]. In the course of neuroinflammation, it can occur by impairing the AQP4 polarity that ensures effective CSF flow [131].

Sleep disorders seem to be inextricably linked to the process of neurodegeneration. This is consistent with the increased incidence of sleep disorders in the elderly and the increasing incidence of neurodegenerative disease in this age group [55]. Chronic sleep disorders are an integral part of neurodegenerative diseases, even being used as a predictor of disease [56]. These disorders, and in RBD, have been shown to cause impairment of both mitochondrial functions and the glymphatic system [57,61]. Furthermore, chronic sleep deprivation, including REM–SD, has been shown to cause abnormalities in mitochondrial morphology and bioenergetics and an increased accumulation of ROS markers, as well as a decreased concentration of numerous antioxidants [62]. Sleep disorders also affect the basic glymphatic system function of regulating CSF flow. The effects of sleep appear to be mainly through affecting cellular energy processes, which are required for the function of Na^+^/K^+^-ATPases [8].

Another factor also linked to the circadian cycle is melatonin concentration. A number of studies have shown that both types of melatonin, pineal and extrapineal, affect the function of the glymphatic system as well as mitochondria [88,89,90,91,92,94]. Key findings include melatonin’s antioxidant effects on neuronal and glial cell mitochondria [95,96,97,98,102], reduction of protein synthesis associated with Aβ formation [107], and increased glymphatic clearance efficiency [108,109].

The text emphasizes the role of sleep disorders in the process of neurodegeneration many times [14,19,20,21,22]. Their potential role as a connector of impaired glymphatic flow and mitochondriopathies was also indicated [15,60,61,62]. The approach to sleep not only as a symptom of neurodegeneration but also as a cause and predictor of this process could bring a new perspective on therapy [58]. For now, melatonin has been shown to appear to be beneficial for the management of RBD [134]. The question remains whether early treatment of sleep disorders will stop or slow down the cascade of events leading to impaired glymphatic clearance and progression of the neurodegeneration process. If this were possible, there remains the question of using a suitably effective therapy to achieve long-term improvement. Another crucial factor in the process of neurodegeneration interacting with the glymphatic system is mitochondrial impairment [41,42,43]. This is already being used as one of the therapeutic targets; for example, zonisamide has been shown to ameliorate microglial mitochondrial dysfunction in a mouse model of Parkinson’s disease [135]. Another therapeutic target connected to the glymphatic system is neuroinflammation, which is used by agomelatine. This drug is an agonist of melatoninergic receptors and is commonly used in depression and sleep disorders occurring in the course of neurodegenerative diseases. In an AD mouse model, its neuroprotective effect was demonstrated. Agomelatine inhibits Aβ deposition and has an anti-inflammatory effect [136]. An approach to alleviating mitochondrial dysfunction and neuroinflammation, which are closely related, is potentially worth considering. Many of the concepts of the therapeutic approaches focus on one element of a complex mechanism. We believe that a simultaneous approach to all of them, with particular emphasis on sleep disorders as early symptoms and one of the major causes of neurodegeneration, can be beneficial.

## 6. Conclusions

The issue of the connection between the glymphatic system and mitochondria in the pathogenesis of neurodegenerative diseases seems multidirectional and complex. Presently it seems impossible to determine the beginning of the cascade of events that seem to lead to the progression of the disease, and this area requires further research. Understanding the mechanism of the relationship between the aforementioned factors could stimulate the development of new therapeutic approaches.

## Figures and Tables

**Table 1:** Sleep disorders in Alzheimer’s and Parkinson’s disease.

Authors	Study Design	Population	Rating Scales and Diagnostic Procedures	Results
McCurry et al., 1999 [19]	Cross-sectional study	205 AD patients, mean age 76.9 years	Mini-Mental State Examination (MMSE), Blessed Dementia Rating Scale (BDRS), The Revised Memory and Behavior Problems Checklist (RMBC), and Physical Examination.	A total of 35% of subjects experienced at least one of seven sleep-related problems during the past week.
Bliwise et al., 1995 [20]	Cross-sectional study	47 AD patients, mean age 80.7 ± 6.5 years	Nightly sleep data based on observations made by nursing staff, Mattis Dementia Rating Scale (DRS), and Katz Activities of Daily Living Scale (ADL).	Patients had moderately disturbed nights of sleep of 24 ± 10% of their nights, and severely disturbed nights of sleep of 7 ± 6% of their nights while staying at an Alzheimer’s disease special care unit.
Zuzuárregui et al., 2020 [22]	Review	PD patients	-	Disorders common in PD: rapid eye movement sleep behavior disorder (RBD), restless legs syndrome, excessive daytime sleepiness, obstructive sleep apnea, and circadian rhythm dysfunction.
Gómez-Esteban et al., 2011 [51]	Cross-sectional study	99 PD patients, mean age 68.5 ± 9.9 years	Quality of life (PDQ-39), Unified Parkinson’s Disease Rating Scale (UPDRS I-IV), Parkinson’s Disease Sleep Scale (PDSS) and Daytime Sleepiness (Epworth), Mini-Mental State Examination, Depression (HAM-D), and the Neuropsychiatric Inventory (NPI-10).	Nighttime sleep disorders such as urinary incontinence, nighttime restlessness, morning fatigue, and somnolence are among the symptoms that significantly affect the quality of life of patients with PD.
Galbiati et al., 2019 [56]	Review	RBD patients	-	A total of 31.95% of RBD patients converted into a neurodegenerative disorder after a mean of 4.75 ± 2.43 years. The most frequent neurodegenerative disorder was represented by PD (44% of converters). Conversion risk of 97% after a follow-up of 14.2 years, and a significantly higher risk for developing PD than for any other.
Bohnen et al., 2019 [58]	Review	PD patients	-	Sleep is common and a major source of disability in PD patients which may modify the course of Parkinson’s disease. The mechanisms by which sleep and neurodegeneration interact are activation of inflammatory pathways, impaired nocturnal brain oxygenation, abnormal proteostasis, and changes in glymphatic clearance. Sleep disturbances may increase the risk of developing PD.
Spira et al., 2013 [49]	Cross-sectional study	Older adults free of cognitive impairment and major diseases; mean age = 76 years.	Self-reported sleep variables, β-Amyloid burden measured by carbon 11-labeled Pittsburgh compound B positron emission tomography distribution volume ratios (DVRs).	Reports of shorter sleep duration and lower sleep quality were associated with greater Aβ burden measured by DVR (*p* = 0.03).
Hahn et al., 2013 [50]	Longitudinal study	A population-based sample of 214 adults aged 75 and over who were dementia-free both at baseline and at first follow-up (3 years later); mean age = 83.4 years at baseline.	At baseline, 40% of participants reported a change in sleep duration. A total of 28.5% were diagnosed with dementia, 22.0% of whom with AD between the 6th and 9th year after baseline.	Reduced sleep was associated with a 75% increased dementia risk (*p* = 0.035) and a 100% increased risk of AD (*p* = 0.019).

## Data Availability

No new data were created or analyzed in this study. Data sharing is not applicable to this article.
